# Essential Oil Volatile Fingerprint Differentiates Croatian cv. Oblica from Other *Olea europaea* L. Cultivars

**DOI:** 10.3390/molecules26123533

**Published:** 2021-06-09

**Authors:** Marijana Popović, Maja Jukić Špika, Maja Veršić Bratinčević, Tonka Ninčević, Ana Matešković, Marija Mandušić, Jakša Rošin, Marija Nazlić, Valerija Dunkić, Elda Vitanović

**Affiliations:** 1 Institute for Adriatic Crops and Karst Reclamation, Put Duilova 11, 21000 Split, Croatia; Marijana.Popovic@krs.hr (M.P.); Maja.Versic.Bratincevic@krs.hr (M.V.B.); Tonka.Nincevic@krs.hr (T.N.); Ana.Mateskovic@krs.hr (A.M.); Marija.Mandusic@krs.hr (M.M.); Jaksa.Rosin@krs.hr (J.R.); Elda.Vitanovic@krs.hr (E.V.); 2 Centre of Excellence for Biodiversity and Molecular Plant Breeding, Svetošimunska Cesta 25, 10000 Zagreb, Croatia; 3 Faculty of Science, University of Split, Ruđera Boškovića 33, 21000 Split, Croatia; marija.nazlic@pmfst.hr (M.N.); valerija.dunkic@pmfst.hr (V.D.)

**Keywords:** *Olea europaea* L., olive leaves, surface area, CIELab color space leaf parameters, chemical characterization, total phenols, essential oil, GC-MS, volatile constituents

## Abstract

Olive leaves are a highly available by-product from table olive and olive oil production. They are nowadays strongly valuable for their major bioactive compounds and their beneficial effects. To determine the differences between two Croatian domestic (Lastovka, Oblica) and two introduced (Leccino, Frantoio) cultivars, physical and chemical analysis of olive leaves were performed: surface area, color variability, total phenolic amounts, and essential oil volatile profiles were analyzed at three harvest periods. All cultivars greatly differed in surface area, with cv. Lastovka being the smallest. Color variability resulted in an overall decrease in darkness and amounts of green and yellow that could be attributed to a decrease in photosynthetic demand and chlorophyll content. The highest amount of total phenolic content occurred in the summer months, followed by a reduction until October. Essential oils volatiles were determined by GC-MS and showed great diversity not only amongst cultivars but also between harvest periods, with overall 45 compounds identified. Principal component analysis distinguished domestic cultivar Oblica from the other observed cultivars, mainly due to its essential oil volatile fingerprint. Compounds that differentiated cv. Oblica were aldehydes ((*E,Z*)-2,4-heptadienal, (*E,E*)-2,4-heptadienal, decanal), ketones ((*E*)-β-damascone, dihydrodehydro-β-ionone), sesquiterpenes (cyclosativene, α-copaene, α-muurolene) and saturated hydrocarbons (tetradecane, hexadecane). Essential oil volatile fingerprint attributed the highest to the biodiversity of domestic cv. Oblica through all three harvest periods.

## 1. Introduction

*Olea europaea* L., is one of the most important and abundant crops in the Mediterranean basin. Mostly it is recognized for its olive fruits as well as for the oil derived from them [1,2]. Considerable amounts of compounds with beneficial health properties are found in both olive fruits and oil, with olive oil being particularly valued as an indispensable part of Mediterranean diet, a healthy balanced diet associated with disease prevention and health maintenance [3,4,5,6].

The olive tree has been used in folk medicine for treating different conditions for centuries [7]. Predominantly olive leaves, but also fruits, seeds, bark, wood, and oil, have been used in various forms to treat diabetes, intestinal diseases, hypertension, asthma, diarrhea, inflammation, and several other conditions. Numerous studies are nowadays performed and the evidence of antimicrobial, antioxidant, antihypertensive and cardioprotective, antidiabetic, anticancer, anti-inflammatory, gastroprotective, and neuroprotective activities of olive tree constituents are growing [8,9,10,11,12,13].

In recent years, there has been a rising interest in studying olive leaves for their major bioactive compounds [14]. Around 10% of the total weight of olives arriving at production mills is attributed to olive leaves, which makes them a highly available by-product from the table olive and olive oil industry [15]. Numerous applications for leaf utilization are considered nowadays [16,17,18]. The leaves are rich in phenolic compounds such as phenolic acids, simple phenols, secoiridoids, flavonoids, lignans, and hydroxy-isochromans, all of which act as natural antioxidants [19]. Oleanolic acid, a pentacyclic triterpenoid with highly valued pharmacological properties, is also found in the leaves [20]. The volatile fractions and essential oils from olive leaves consist of several valuable classes of chemical compounds that contribute to its beneficial health effects; mainly aldehydes, terpenoids, alcohols, and ketones, whereas they show antioxidant activity as well as antimicrobial and antifungal activity [21]. The addition of olive leaves to olive oil improves its nutraceutical properties. The phenolic compounds from leaves add to the overall phenolic content of olive oil and their health benefits, as well as in the enhancement of the antioxidant capacity of olive oil. Volatile compounds found in essential oil could also contribute to additional nutraceutical and sensorial features [22,23]. Furthermore, leaf volatiles are very important in the interaction of plants with the environment, as they are involved in the protection against biotic stressors as well as against abiotic stress [24,25].

The aim of this study was to determine differences in surface area, color, total phenolic amounts, and essential oil volatile profiles of olive leaves at different harvesting periods in order to investigate biodiversity amongst domestic Croatian and introduced cultivars. Four cultivars, two domestic cultivars Lastovka and Oblica, and two introduced cultivars Leccino and Frantoio were analyzed. Domestic cultivars Oblica and Lastovka are the most present cultivars in this region and of high local importance, whilst introduced cvs. Leccino and Frantoio are some of the most present olive cultivars in Mediterranean olive regions and serve as a good reference model in the distinguishment of domestic from introduced cultivars. All four cultivars are grown in the same olive orchard with the same environmental conditions, hence all of the differences would be attributed solely to the studied cultivar. To the best of our knowledge, this is the first study on essential oils in olive leaves conducted using Oblica and Lastovka cultivars from the Croatian part of Mediterranean basin.

## 2. Results and Discussion

### 2.1. Surface Area and Color Variability

**Figure 1:**
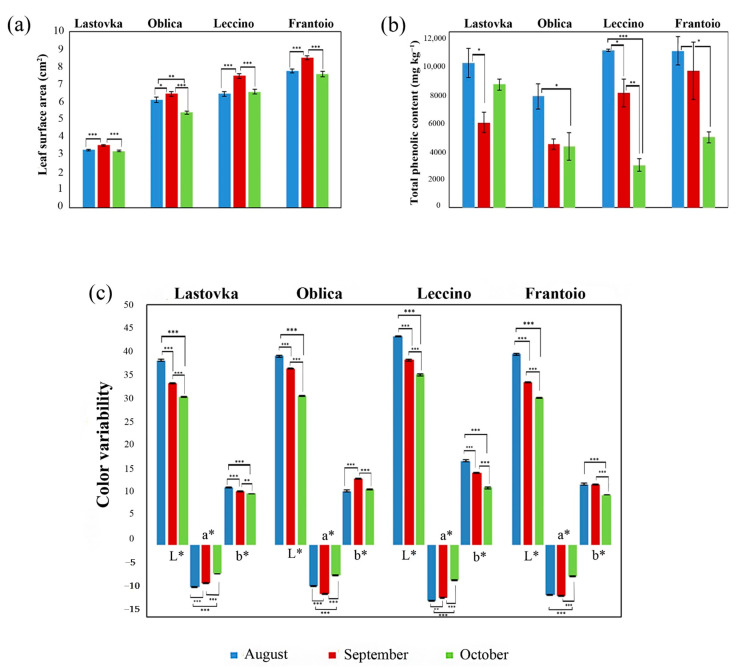
Graphical representation of the values obtained for surface area, total phenolic amount and color variability of leaves from cvs. Lastovka, Oblica, Leccino and Frantoio at different harvest periods. Statistically significant test result is considered for *p* ≤ 0.05. * *p* ≤ 0.05; ** *p* ≤ 0.01; *** *p* ≤ 0.001. (**a**) Difference in leaf surface area from cvs. Lastovka, Oblica, Leccino and Frantoio at 22 August, 12 September, and 25 October (**b**) Difference in total phenolic content from cvs. Lastovka, Oblica, Leccino and Frantoio at 22 August, 12 September, and 25 October (**c**) Difference in color variability from cvs. Lastovka, Oblica, Leccino and Frantoio at 22 August, 12 September, and 25 October.

Physical characteristics were observed in all four cultivars through the measurements of surface area and color variability (Figure 1). There were significant differences amongst cultivars at each harvest period examined: cv. Oblica showed a significant difference in surface area at each individual harvest period while cvs. Lastovka, Leccino, and Frantoio differed significantly between harvests of August and September, as well as between September and October (Table S1). Leaf surface area increased from August to September, followed by a decrease in October, following a similar pattern from a previous study on Portuguese cultivars [26]. The leaves of cv. Frantoio were the largest and varied between 7.57 and 8.52, while the leaves of cv. Lastovka were the smallest and varied between 3.22 and 3.55 cm^2^.

Leaf color variability was measured for three parameters: lightness (L*), greenness/redness (a*), and blueness/yellowness (b*). The parameter L* indicating lightness/darkness of the leaves was highest (43.37 ± 0.15) in cv. Leccino in the first harvest. Cv. Leccino was also the most negative cultivar for a* (−11.64 ± 0.09) indicating the highest level of green, and most positive for b* (17.48 ± 0.17) indicating the highest level of yellow (Figure 1). The parameters L*, a*, and b* decreased in all cultivars from August to October with the exception of cv. Oblica for a* and b* which increased in September and decreased in October, as well as cv. Frantoio following the same pattern for b*. Malheiro et al. reported a similar pattern of changes in color values for different cultivars [26]. The overall reduction in darkness and the amounts of green and yellow could be attributed to the reduction in metabolic demand of olive leaves due to fruit ripening, resulting in a decrease in photosynthetic demand and chlorophyll content. The results are in accordance with those of Proietti et al. who showed that chlorophyll content decreased during the same period in cvs. Leccino and Frantoio [27].

### 2.2. Total Phenols

Cultivar Leccino showed significant statistical difference at each harvest period (Table S1) and also had the highest amount of total phenols (August, ±108 mg kg^−1^) among all cultivars (Figure 1), which decreased sharply until October (3025 ± 434 mg kg^−1^). The amounts of total phenols in cvs. Frantoio and Oblica also decreased from August until October, with a significant difference only between August and October (Figure 1, Table S1). The lowest amount of total phenols was observed in domestic cv. Oblica (August, 7902 ± 880 mg kg^−1^). Cultivar Lastovka also showed an overall decrease in total phenol amount; however, it differed from other cultivars in the pattern of total phenol change showing a significant decrease from August until September, followed by an increase of total phenols till October. The concentration of phenolic compounds in olive leaves was shown to be highest in the summer months, indicating that the influence of temperature directly affects the synthesis of phenolic compounds [28,29]. Our data for cvs. Leccino and Frantoio are in accordance with previous research by Blasi et al. in which the amount of total phenols decreases from summer to autumn months [30]. Several studies have been carried out for the phenolic amounts in cvs. Lastovka and Oblica; however, none of them were carried out at the same harvest periods [31,32,33].

### 2.3. Essential Oil Volatiles

The results of the volatile profiles of olive leaves essential oil of four different olive cultivars Lastovka, Oblica, Leccino and Frantoio in three harvest periods are presented in Table 1 and Table 2, Figures S1–S4. Statistical differences between cultivars and harvest periods are shown in Table S2. Overall, 45 volatile compounds were identified in essential oils, ranging from 93.46 (cv. Oblica) to 98.21% (cv. Frantoio) of the total identified compounds from the chromatograms. The identified volatile compounds include two alcohols, eight aldehydes, six ketones, two aromatic hydrocarbons, three esters, two saturated hydrocarbons, five monoterpenes, eleven sesquiterpenes, five heterocyclic compounds and a pyridine.

The main components of the essential oil from olive leaves of all four cultivars collected in August, September and October belonged to the ketone group, with (*E*)-β-damascenone (23.78% for cv. Frantoio to 36.77% for cv. Leccino) being a major constituent with the exception of nonanal (24.80%) for cv. Leccino harvested in October and β-caryophyllene (16.65%) for cv. Lastovka in September. Previous studies on essential oils of cvs. Leccino and Frantoio conducted at different harvest periods in Italy also showed high percentage of ketones, (*E*)-β-damascenone in particular [34,35]. A higher presence of dihydrodehydro-β-ionone was detected for cv. Oblica in first and second harvest. Overall, regardless of the olive cultivar, ketones were the most represented chemical class, differentiating from 20.49% for cv. Lastovka harvest in September to 52.05% for cv. Leccino harvest in August. The identified ketones were 1,4-dimethyl-δ-3-tetrahydroacetophenone, (*E*)-β-damascenone, (*E*)-β-damascone, dihydrodehydro-β-ionone, (*E*)-geranyl acetone and β-ionone. Through our study, a significant decrease in ketones was observed in all cultivars except in cv. Lastovka, with a decrease in September and an increase in October.

Only two alcohols were identified in all cultivars, (*Z*)-3-hexen-1-ol, which was present in all harvest periods of all cultivars, in contrast to octanol, which was present in smaller amounts and was not identified in all harvest periods for all four cultivars studied. (*Z*)-3-hexen-1-ol showed an increase in all cultivars, except in cv. Lastovka, where there was a decrease in all harvest periods. In olive fruits, (*Z*)-3-hexen-1-ol is one of the aroma compounds largely contributing to the first stage of ripeness as well as the over-ripe stage [38]. It is also well known green leaf volatile attributing to ‘green odor’ of leaves [39].

Aldehydes were present in a significant amount ranging from 9.34% for cv. Frantoio harvested in September to 34.79% for cv. Leccino leaves samples harvested in October, in which they formed the most abundant chemical class identified. The presence of eight aldehydes was confirmed, heptanal, benzaldehyde, (*E*,*Z*)-2,4-heptadienal, (*E*,*E*)-2,4-heptadienal, phenylacetaldehyde, nonanal, decanal and (E)-2-decenal, whereas nonanal particularly stood out, showing the largest amount in all cultivars. Campeol et al. also identified all these aldehydes, with nonanal as the most abundant [34,35].

Heterocyclic compounds were present in a considerable amount, with a total of five compounds identified. The most prominent was theaspirane a in all four cultivars, with the largest percentage in cv. Lastovka (9.17–12.21%) and in cv. Frantoio (10.68–13.54%). Vitispirane, dihydroedulan I and II were found in a slightly lower amount being the most abundant in cv. Lastovka, ranging from 0 to 1.37%.

Only two identified compounds belong to a group of saturated hydrocarbons: tetradecane and hexadecane, with tetradecane present in a greater amount. Cv. Oblica is particularly rich in these compounds, with over 20% in August harvest. A decrease in the concentration of saturated hydrocarbons was observed in all cultivars except in cv. Frantoio through all three harvest periods.

Eleven sesquiterpenes have been identified, with β-caryophyllene particularly standing out as it appeared in very high amounts in the second harvest (September) in cv. Lastovka with 16.65%, increasing from 4.94% after the August harvest and decreasing to 10.23% in the last harvest in October. The lowest amount of β-caryophyllene was recorded in all harvest periods for the Oblica cultivar, while an increase followed by a decrease was observed in cvs. Leccino and Frantoio; somewhat more significantly in the Frantoio cultivar. α-copaene showed the highest percentage of all sesquiterpenes for the Oblica cultivar, with an increase in all three harvest periods, from 6.10% in August to 9.06% and 11.14% in September and October, respectively. α-farnesene was detected in low amounts in all cultivars, except in the cv. Oblica. Sesqiterpenes are a wide group of secondary metabolites that raise attention in the bio-pharmacological field, especially as potential natural anticancer compounds [40]. Among them, β-caryophyllene is intensively studied for its countless biological properties [41].

As for the chemical class of esters, methyl salicylate, chrysanthenyl acetate and (*E*)-2-hexenyl benzoate were identified. Over time chrysanthenyl acetate decreased in cvs. Lastovka and Oblica while an increase occurred in Leccino and Frantoio cultivars. (*E*)-2-hexenyl benzoate increased slightly in cv. Frantoio, and was absent in Lastovka and Oblica cultivars, as well as in the third harvest for cv. Leccino.

Monoterpenes represent a smaller fraction of volatiles in the studied cultivars. Five monoterpenes, cis-linalool oxide, trans-linalool oxide, limonen-4-ol, α-terpineol and β-cyclocitral were identified in all essential oils. Cis-linalool oxide was detected in all cultivars except in cv. Frantoio while trans-linalool oxide was present only in domestic cultivars.

Aromatic hydrocarbons, p-cymenene and α-ionene, represent the group of chemical compounds with the lowest percentage in all analyzed cultivars. α-ionene was present in a slightly higher amount and showed a significant difference in cv. Lastovka and cv. Leccino through all harvest periods. Cvs. Oblica and Leccino had a significant difference in the amount of p-cymenene between the September and October harvests.

Only one pyridine, pyridine-3-ethnyl, was identified. Small amounts of pyridine-3-ethnyl were found in cv. Lastovka in all periods (0.32–1.01%); in Oblica and Frantoio cultivars it was identified only in September, while in cv. Leccino in September and October.

### 2.4. Principal Component Analysis

**Figure 2:**
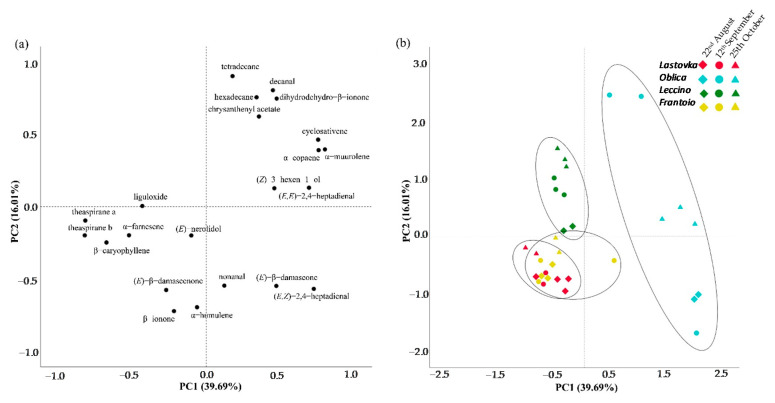
Principal component analysis (PCA) of the volatiles in the amount larger than 2% from cvs. Lastovka, Oblica, Leccino and Frantoio at different harvest periods. (**a**) PCA loading plots of volatiles from the first and second principal component; (**b**) PCA score plot allocating different cultivars into clusters.

To summarize the data obtained from the physical and chemical analysis, we subjected the values for surface area, color variability, total phenols and the volatiles in the amount larger than 2% to a principal component analysis. In order to furthered investigate our data, we performed additional PCAs separately for physical and chemical data, as well as for volatile compounds in the amount larger than 2% alone, to ask whether the volatile fingerprint of different cultivars per se can be a differentiating factor (Figures S5–S7, Table S3). The first two PCAs from combined data of physical and chemical analysis explained 48.97% of the variance and distinguished cv. Oblica from the rest of the cultivars. The PCA performed only for the physical characteristics distinguished cv. Lastovka from the other three cultivars studied, which was expected since the highest of total four obtained communalities was for the surface area 0.978 (Table S3), and even more so since the surface area of cv. Lastovka was half that of the other cultivars studied (Figure 1). The most interesting results were provided by PCA performed for volatile compounds where the first two PCAs explained 55.69% of variance and clearly differentiated volatile fingerprint of essential oil of cv. Oblica from cv. Lastovka and both introduced cultivars in all harvest periods (Figure 2). In a recent study Pasković et al. tried to differentiate cultivars Oblica, Lastovka and Leccino, as well as cv. Drobnica not according to their volatiles but according to main phenolic components, and were able to distinguish only cv. Drobnica on that behalf [33].

**Figure 3:**
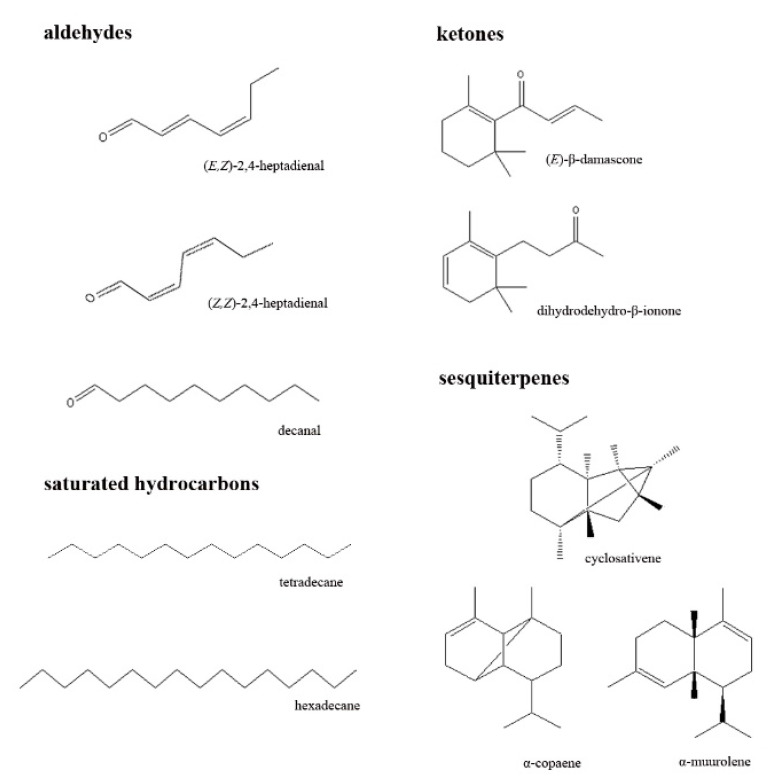
Volatile compounds responsible for differentiation of cv. Oblica from other analyzed olive leaves cultivars.

Although cv. Oblica is well distinguished in PCA in which physical and chemical analysis were included, more variance is explained in the first two PCAs for volatile compounds only, revealing the important role they play in profiling and discriminating between cultivars. The components differentiating cv. Oblica are located in the positive region of PC1 and both positive and negative regions of PC2. The volatiles presented in a higher amount that characterize cv. Oblica are mainly aldehydes ((*E*,*Z*)-2,4-heptadienal, (*E*,*E*)-2,4-heptadienal, decanal), ketones ((*E*)-β-damascone, dihydrodehydro-β-ionone), sesquiterpenes (α-copaene, cyclosativene, α-muurolene) and saturated hydrocarbons (tetradecane, hexadecane) (Figure 3). α-copaene, the most abundant sesquiterpene from cv. Oblica, was recently studied and showed concentration-dependent cytotoxic effect as well as antioxidant capacity for human lymphocytes cells [42].

Unlike cv. Oblica, the Leccino cultivar also differs well from the other cvs. in the September and October harvests, while the pattern of volatiles in August does not differ from that of cv. Frantoio in October. Cvs. Lastovka and Frantoio exceptionally overlap and cannot be differentiated in any harvest period.

## 3. Materials and Methods

### 3.1. Plant Material

Plant material was sampled in the experimental olive orchard of Institute for Adriatic Crops and Karst Reclamation in Kaštel Stari, Croatia (43°55′ N; 16°35′ E, 28 m above sea level). Fresh leaves were collected at three harvests, 22 August, 12 September, and 25 October from four cultivars: Leccino, Frantoio, Lastovka, and Oblica. For morphological and analytical measurements, the leaves samples were collected according to methodology defined by Barranco and Rallo [43], adult leaves were taken from the middle section of 8-10 one-year-old shoots selected from the most representative shoots on the south-facing side of the tree at shoulder level.

### 3.2. Leaf Surface Area

To determine the adaxial leaf surface area values, forty olive leaves [43] per replicate x three replicates were scanned by an Epson Perfection V700 Photo scanner (Konica, Tokyo, Japan). The obtained scans of olive leaves were processed by WinFOLIA Pro 2014a 32-bit analysis program.

### 3.3. Leaf Color

Color analysis was performed on the same leaves samples of cvs. Lastovka, Oblica, Leccino and Frantoio on which dimensional measurements were determined by Konica Minolta Chroma Meter CR-400 instrument (Konica, Tokyo, Japan). Two measurements were made at the adaxial surface area of each leaf. Monochromatic variables L*, a* and b* were measured to determine color space co-ordinates: L* as a measure of lightness, a* as a measure of greenness/redness, and b* for blueness/yellowness. The colorimeter was calibrated against a standard white plate before leaf color measurement [44].

### 3.4. Determination of Total Phenols

#### 3.4.1. Extraction

The plant material was freeze-dried (FreeZone 2.5, Labconco, Kansas City, MO, USA) and a sample of the dry olive leaves was ground to a coarse powder using a stainless steel mill (A 11 Analytical mill, IKA, Staufen, Germany). For the extraction of phenols procedure described by Marinova et al. [45] was followed. Briefly: 0.5 g of powdered tissue was extracted with 50 mL of methanol/water (80:20, by volume) during 20 min on an ultrasonic bath (Sonorex Digitec DT 100H, Bandelin, Berlin, Germany). An aliquot was centrifuged for 5 min at 14,000 rpm (Beckman Instruments J2-21, Palo Alto, CA, USA).

#### 3.4.2. Determination of Total Phenolic Content

The total phenolic content was determined according to the Folin–Ciocalteu method [46] with slight modifications: leaves extract (0.5 mL) was mixed with distilled deionized water (9 mL) and Folin–Ciocalteu reagent (1mL) (Sigma-Aldrich, Steineheim, Germany). Sodium carbonate (Kemika, Zagreb, Croatia) (7 g/100 mL) was added five minutes after and filled with dd H_2_O up to a final volume of a 25-mL volumetric flask. Quantification was done by measuring the absorbance at 470 nm (Cary 50 UV-VIS spectrophotometer; Varian, CA, USA) after 90 min of reaction at room temperature against a prepared blank. All measurements were performed in triplicate and results were expressed as milligrams of gallic acid equivalents per gram of leaf (dry weight).

### 3.5. Determination of Volatiles

#### 3.5.1. Essential Oil Distillation

Approximately 250 g of fresh leaves per sample were placed to air dry at room temperature (22 °C) for 15 days. A hundred grams of dried leaves per sample were chopped and then hydro-distilled in a Clevenger apparatus for two hours and 30 min. Three replicate samples were distilled simultaneously. Pentane and diethyl-ether (VWR, Radnor, PA, USA) were used as solvents. The oil samples were dried over anhydrous sodium sulfate (Kemika, Zagreb, Croatia) and then stored in glass vials at 4 °C until further analysis.

#### 3.5.2. Gas Chromatography-Mass Spectrometry

Gas chromatography-mass spectrometry (GC-MS) analysis of the essential oils was performed using a gas chromatograph (model 3900, Varian Inc., Lake Forest, CA, USA) and mass spectrometer (model 2100T, Varian Inc., Lake Forest, CA, USA). A non-polar capillary column VF-5ms (length 30 m, inside diameter 0.25 mm, coating thickness 0.25 µm, Palo Alto, CA, USA) was used for chromatography. Analyses were performed using MS full scan (40–350 *m*/*z*). The initial column temperature of 60 °C was held for 3 min, and then increased to 246 °C at a rate of 3 °C min^−1^, and kept isothermal for 25 min [47]. The injection volume was 2 µL, and the split ratio was 1:20. Ultra-pure helium was used as the carrier gas with flow rate set to 1.5 mL min^−1^. The ion source temperature was set at 200 °C and the ion voltage was 70 eV. Identification of individual peaks was made by comparison of their retention indices with the series of n-hydrocarbons, along with the computer matching of mass spectra with commercial databases (NIST 98 and Wiley 7) and by comparison with literature data [34,35,36,37]. All the analyses were performed in triplicate and expressed as mean ± SE of component percentage.

### 3.6. Statistical Analysis

#### 3.6.1. Analysis of Variance

To determine the difference in surface area, color, total phenols, and essential oil volatiles in the three harvest periods, one-way analysis of variance (ANOVA) was performed by SPSS software, version 25.0 (IBM Corporation, New York, NY, USA). First, the data were tested for normal distribution of the residuals as well as for the homogeneity of variance by Shapiro–Wilk’s test. If the requirement of homogeneity was fulfilled, one-way ANOVA was performed; otherwise, Welch correction was preceded first. If there was a statistically significant effect between different harvest periods, Tukey’s honestly significant test for equal variances or Dunnett T3 test for non-equal variances was performed, at the significance level of *p* ≤ 0.05.

#### 3.6.2. Principal Component Analysis

In order to summarize the data and reduce the number of variables determined by physical and chemical analysis, the values of surface area, color, total phenols and essential oil volatiles in the amount larger than 2% from all cultivars at different harvest periods were subjected to principal component analysis (PCA). To further investigate our results, additional PCAs were performed separately for the physical and chemical analysis data, as well as for essential oil volatiles in the amount larger than 2%. The Kaiser–Meyer–Olkin measure of sampling adequacy was performed before the analysis to see if factor analysis is suitable for our data. To determine whether the factors could allocate different olive cultivars into the clusters, a two-dimensional score plot was performed. Varimax with Kaiser Normalization was used for the rotation method in the rotated component matrix. PCAs were performed using SPSS software, version 25.0 (IBM Corporation, New York, NY, USA).

## 4. Conclusions

The results of physical and chemical analysis of olive leaves from two Croatian domestic (Lastovka, Oblica) and two introduced (Leccino, Frantoio) cultivars from three harvest periods showed great diversity amongst cultivars. Leaf surface area was the most significant physical trait that distinguished cv. Lastovka from other cultivars. Color variability and total phenolic content showed a similar pattern amongst all cultivars, while essential oil volatiles clearly distinguished cv. Oblica from other cultivars. Various aldehydes, ketones, sesquiterpenes and saturated hydrocarbons made the greatest contribution in the differentiation of cv. Oblica from other cultivars, much more than any other parameter analyzed. Through all three harvest periods, cv. Oblica could be differentiated based on its essential oil volatile fingerprint.

## Figures and Tables

**Table 1:** Composition of volatiles identified at different harvest periods in essential oils of olive leaves from cvs. Lastovka and Oblica.

Compound	KI	Lastovka			Oblica		
		22 August	12 September	25 October	22 August	12 September	25 October
(*Z*)-3-hexen-1-ol	849	3.51 ± 0.19 _a_	2.32 ± 0.05 _a_	1.55 ± 0.2	3.46 ± 0.47	7.30 ± 1.17	3.56 ± 0.73
heptanal	902	-	-	0.15 ± 0.0004	-	0.93 ± 0.03	1.17 ± 0.13
benzaldehyde	977	1.10 ± 0.02 _a,b_	0.35 ± 0.04 _a_	0.39 ± 0.003 _b_	1.47 ± 0.86	0.78 ± 0.19	0.85 ± 0.25
pyridne-3-ethnyl	986	1.01 ± 0.1 _a,b_	2.87 ± 0.45 _a,c_	0.32 ± 0.01 _b,c_	-	0.49 ± 0.09	-
(*E,Z*)-2,4-heptadienal	1008	0.92 ± 0.05	1.04 ± 0.05	1.02 ± 0.09	0.66 ± 0.18 _a,b_	4.66 ± 1.17 _a_	2.08 ± 0.19 _b_
(*E,E*)-2,4-heptadienal	1027	0.54 ± 0.02	0.4 ± 0.03	0.96 ± 0.17	1.53 ± 0.34	2.38 ± 0.98	3.83 ± 1.16
phenylacetaldehyde	1063	1.57 ± 0.30	0.79 ± 0.06	0.48 ± 0.19	0.66 ± 0.09	1.82 ± 0.47	1.46 ± 0.35
cis-linalool oxide (furanoid)	1079	0.27 ± 0.02 _a_	0.11 ± 0.01 _a_	tr	1.36 ± 0.08 _b_	0.77 ± 0.34	0.79 ± 0.05 _b_
octanol	1087	0.46 ± 0.02 _a_	0.68 ± 0.05 _a_	0.66 ± 0.10	-	0.29 ± 0.07	-
trans-linalool oxide (furanoid)	1092	0.25 ± 0.03	0.18 ± 0.02	-	0.72 ± 0.03	0.71 ± 0.22	0.59 ± 0.04
p-cymenene	1100	0.33 ± 0.02	0.35 ± 0.01	0.19 ± 0.04	-	0.37 ± 0.05 _c_	0.15 ± 0.03 _c_
nonanal	1107	8.00 ± 0.39	6.65 ± 0.57	7.94 ± 0.65	1.98 ± 0.35 _a,b_	7.35 ± 0.06 _a_	6.15 ± 1.89 _b_
1,4-dimethyl-δ-3-tetrahydroacetophenone	1158	1.63 ± 0.12	1.69 ± 0.03	1.46 ± 0.09	0.52 ± 0.04 _a_	1.81 ± 0.13 _a,c_	0.63 ± 0.13 _c_
limonen-4-ol	1193	1.44 ± 0.19	1.77 ± 0.14	1.86 ± 0.12	1.11 ± 0.15	0.97 ± 0.18	1.13 ± 0.23
decanal	1200	0.68 ± 0.05 _b_	0.57 ± 0.02 _c_	0.37 ± 0.003 _b,c_	4.12 ± 1 _a_	0.98 ± 0.38 _a,c_	3.09 ± 0.25 _c_
α-terpineol	1210	0.85 ± 0.08	0.86 ± 0.11	1.34 ± 0.09	1.30 ± 0.6	0.36 ± 0.29	0.18 ± 0.09
methyl salicylate	1210	0.51 ± 0.07 _a_	1.22 ± 0.13 _a,c_	0.29 ± 0.03 _c_	0.5 ± 0.27	1.63 ± 0.41	1.62 ± 0.73
β-cyclocitral	1228	0.76 ± 0.06	0.58 ± 0.04 _c_	0.85 ± 0.04 _c_	0.57 ± 0.14	1.47 ± 0.11	0.53 ± 0.11
chrysanthenyl acetate	1243	1.87 ± 0.23	1.95 ± 0.14	1.67 ± 0.22	3.01 ± 0.88	1.52 ± 0.3	2.95 ± 0.51
α-ionene	1262	0.63 ± 0.05 _b_	0.64 ± 0.02 _c_	0.44 ± 0.04 _b,c_	1.66 ± 0.52	0.87 ± 0.03	0.73 ± 0.19
(*E*)-2-decenal	1276	0.28 ± 0.02 _a,b_	0.58 ± 0.09 _a_	0.76 ± 0.07 _b_	-	tr	tr
vitispirane	1283	1.27 ± 0.07 _a_	0.93 ± 0.02 _a_	1.19 ± 0.10	0.12 ± 0.03 _a_	0.33 ± 0.05 _a_	0.19 ± 0.02
dihydroedulan II	1294	-	-	tr	-	0.14 ± 0.04	0.11 ± 0.02
dihydroedulan I	1299	1.37 ± 0.11	1.0 ± 0.02 _c_	1.37 ± 0.01 _c_	-	-	tr
theaspirane a ^#^	1303	12.21 ± 0.57 _a_	9.17 ± 0.36 _a_	11.12 ± 0.39	2.29 ± 0.12	3.6 ± 0.7	2.44 ± 0.38
theaspirane b ^#^	1321	9.81 ± 0.29 _a_	7.8 ± 0.09 _a,c_	9.02 ± 0.26 _c_	1.69 ± 0.04 _a_	3.5 ± 0.69 _a_	1.83 ± 0.31
cyclosativene	1373	0.13 ± 0.01 _b_	0.12 ± 0.002 _c_	0.18 ± 0.02 _b,c_	1.59 ± 0.11	2.05 ± 0.12	2.4 ± 0.57
α-copaene	1382	0.45 ± 0.06 _b_	0.49 ± 0.02 _c_	0.84 ± 0.11 _b,c_	6.10 ± 0.79	9.06 ± 0.56	11.14 ± 3.16
(*E*)-β-damascenone	1384	15.00 ± 0.47 _a_	11.99 ± 0.49 _a_	14.67 ± 1.04	6.85 ± 1.89	12.26 ± 1.73	5.77 ± 1.97
tetradecane	1400	7.60 ± 0.28 _a,b_	5.96 ± 0.18 _a,c_	4.17± 0.28 _b,c_	15.47 ± 1.33 _a,b_	3.27 ± 0.62 _a,c_	9.41 ± 0.66 _b,c_
(*E*)-β-damascone	1417	0.97 ± 0.12 _b_	1.00 ± 0.04 _c_	1.77 ± 0.07 _b,c_	1.05 ± 0.18	2.62 ± 0.53	1.17 ± 0.33
β-caryophyllene	1427	4.94 ± 0.67 _a,b_	16.65 ± 1.44 _a_	10.23 ± 0.83 _b_	-	1.49 ± 0.23	1.94 ± 0.17
dihydrodehydro-β-ionone	1431	4.21 ± 0.56 _a_	2.54 ± 0.18 _a_	2.8 ± 0.03	22.53 ± 2.67 _a_	5.28 ± 0.51 _a,c_	13.29 ± 2.22 _c_
(*E*)-geranyl acetone	1461	tr	0.20 ± 0.02 _c_	0.56 ± 0.07 _c_	-	-	-
α-humulene	1464	0.85 ± 0.06 _a,b_	2.13 ± 0.30 _a_	1.75 ± 0.33 _b_	-	1.33 ± 0.41	1.23 ± 0.19
β-ionone	1488	3.84 ± 0.2	3.07 ± 0.24 _c_	4.65 ± 0.28 _c_	0.91 ± 0.16 _a,b_	5.25 ± 0.79 _a_	2.61 ± 0.49 _b_
α-muurolene	1512	-	tr	tr	1.63 ± 0.18	2.32 ± 0.07	2.60 ± 0.68
α-farnesene	1514	1.08 ± 0.32 _a,b_	3.02 ± 0.48 _a_	3.38 ± 0.30 _b_	-	-	-
δ-cadinene	1527	0.44 ± 0.06 _b_	0.33 ± 0.03	0.22 ± 0.01 _b_	0.18 ± 0.08	0.49 ± 0.01	0.30 ± 0.08
kessane	1542	tr	tr	tr	-	-	tr
liguloxide	1547	0.97 ± 0.05 _b_	1.08 ± 0.04 _c_	1.91 ± 0.11 _b,c_	0.51 ± 0.1 _b_	0.57 ± 0 _c_	1.37 ± 0.17 _b,c_
(*E*)-nerolidol	1575	0.92 ± 0.03 _a,b_	1.56 ± 0.10 _a,c_	2.88 ± 0.05 _b,c_	1.05 ± 0.21	1.11 ± 0.23	1.12 ± 0.15
caryophyllene oxide	1594	1.52 ± 0.05	1.64 ± 0.21	1.07 ± 0.05	1.62 ± 0.38	0.57 ± 0.19	1.12 ± 0.11
(*E*)-2-hexenyl benzoate	1599	-	-	-	-	-	-
hexadecane	1606	0.89 ± 0.05	0.52 ± 0.14	0.44 ± 0.05	5.23 ± 0.49 _b_	0.83 ± 0.22 _b,c_	3.51 ± 0.77 _c_
	Alcohols	3.97	3.00	2.2	3.46	7.59	3.56
	Aldehydes	13.86	10.95	12.93	10.99	20.48	19.19
	Ketones	25.73	20.49	25.91	31.87	27.21	23.47
	Esters	2.39	3.16	1.97	3.51	3.15	4.56
	Aromatic hydrocarbones	0.96	0.99	0.63	1.66	1.24	0.87
	Saturated hydrocarbons	8.50	6.48	4.61	20.70	4.10	12.91
	Monoterpenes	3.57	3.50	4.12	5.06	4.28	3.23
	Sesquiterpenes	11.36	27.10	22.60	12.67	19.00	23.32
	Heterocyclic compounds	24.66	18.92	22.71	4.10	7.52	4.65
	Pyridines	1.01	2.87	0.32	0.00	0.49	-
Total identified (%)		95.22	96.88	97.14	93.46	93.60	95.25

Results are expressed as mean ± SE. Kovats indices (KI) are determined on the VF-5MS capillary column; -, not detected; tr, traces (<0.01%). Compounds are identified by mass spectra and KI comparison with NIST and Wiley libraries, as well as literature values [34,35,36,37]. Statistical analyses are performed by one-way ANOVA analysis, with or without Welch corrections. Means were compared by Tukey’s test if equal variance could be assumed or by Dunett T3’s test if equal variance could not be assumed. Mean values with the same letter differ significantly (*p* ≤ 0.05). theaspirane ^#^–correct isomer could not be determined.

**Table 2:** Composition of volatiles identified at different harvest periods in essential oils of olive leaves from cvs. Leccino and Frantoio.

Compound	KI	Leccino			Frantoio		
		22 August	12 September	25 October	22 August	12 September	25 October
(*Z*)-3-hexen-1-ol	849	1.32 ± 0.35	2.13 ± 0.17	1.61 ± 0.24	3.87 ± 0.25	2.50 ± 0.17	4.11 ± 0.53
heptanal	902	0.31 ± 0.02 _a,b_	0.48 ± 0.05 _a,c_	0.82 ± 0.04 _b,c_	0.30 ± 0.06	0.22 ± 0.22	0.55 ± 0.1
benzaldehyde	977	0.23 ± 0.002	0.28 ± 0.04	0.40 ± 0.03	0.35 ± 0.05 _b_	0.50 ± 0.05 _c_	0.89 ± 0.05 _b,c_
pyridne-3-ethnyl	986	-	1.78 ± 0.23	0.59 ± 0.32	-	3.43 ± 0.21	-
(*E,Z*)-2,4-heptadienal	1008	1.18 ± 0.23	2.47 ± 0.18	3.15 ± 0.04	1.23 ± 0.19	1.05 ± 0.19	1.98 ± 0.24
(*E,E*)-2,4-heptadienal	1027	0.74 ± 0.1 _a,b_	1.19 ± 0.09 _a,c_	1.91 ± 0.07 _b,c_	0.54 ± 0.08 _b_	0.5 ± 0.002 _c_	2.81 ± 0.56 _b,c_
phenylacetaldehyde	1063	0.84 ± 0.01	1.62 ± 0.27	1.11 ± 0.12	1.24 ± 0.15	1.73 ± 0.09	1.23 ± 0.27
*cis*-linalool oxide (furanoid)	1079	0.16 ± 0.02	-	tr	-	-	-
octanol	1087	1.09 ± 0.1	0.92 ± 0.13	0.98 ± 0.07	0.51 ± 0.06 _b_	0.96 ± 0.03 _c_	0.17 ± 0.03 _b,c_
*trans*-linalool oxide (furanoid)	1092	-	-	-	-	-	-
p-cymenene	1100	0.24 ± 0.01	0.16 ± 0.01 _c_	0.25 ± 0.03 _c_	0.26 ± 0.04	tr	tr
nonanal	1107	6.08 ± 0.87 _b,c_	7.46 ± 1.26 _c_	24.80 ± 2.85 _b_	7.88 ± 0.67 _a_	3.67 ± 0.28 _a,c_	11.15 ± 2.47 _c_
1,4-dimethyl-δ-3-tetrahydroacetophenone	1158	0.97 ± 0.14	1.21 ± 0.14 _c_	0.70 ± 0.06 _c_	1.15 ± 0.23	0.71 ± 0.17	0.62 ± 0.07
limonen-4-ol	1193	0.79 ± 0.16	0.81 ± 0.14	0.73 ± 0.02	0.61 ± 0.17	0.39 ± 0.07	1.09 ± 0.14
decanal	1200	0.88 ± 0.15	0.69 ± 0.03	0.66 ± 0.1	0.76 ± 0.09	0.64 ± 0.57	1.36 ± 0.43
α-terpineol	1210	0.81 ± 0.29	1.52 ± 0.26	0.86 ± 0.01	0.22 ± 0.07	0.39 ± 0.07	0.56 ± 0.09
methyl salicylate	1210	0.49 ± 0.18	0.42 ± 0.04	0.65 ± 0.09	1.01 ± 0.09	0.58 ± 0.07	0.32 ± 0.09
β-cyclocitral	1228	0.71 ± 0.06 _a_	0.95 ± 0.02 _a_	0.81 ± 0.02	0.71 ± 0.03	0.84 ± 0.16	0.58 ± 0.08
chrysanthenyl acetate	1243	1.05 ± 0.23	1.23 ± 0.16	1.23 ± 0.1	0.67 ± 0.26	0.63 ± 0.29	1.32 ± 0.24
α-ionene	1262	1.14 ± 0.01 _a,b_	0.50 ± 0.02 _a_	0.57 ± 0.1 _b_	0.27 ± 0.12	0.14 ± 0.004	0.18 ± 0.03
(*E*)-2-decenal	1276	0.57 ± 0.09 _b_	0.68 ± 0.04 _c_	1.14 ± 0.09 _b,c_	0.58 ± 0.18	0.19 ± 0.03	0.15 ± 0.06
vitispirane	1283	0.72 ± 0.09 _a_	0.49 ± 0.01	0.45 ± 0.04 _a_	1.11 ± 0.08 _a,b_	0.67 ± 0.05 _a_	0.55 ± 0.05 _b_
dihydroedulan II	1294	0.24 ± 0.02	0.29 ± 0.03	0.22 ± 0.02	0.12 ± 0.02	tr	tr
dihydroedulan I	1299	0.51 ± 0.01 _b_	0.38 ± 0.03 _c_	0.27 ± 0.02 _b,c_	0.85 ± 0.04 _a_	0.28 ± 0.11 _a_	0.51 ± 0.06
theaspirane a ^#^	1303	3.60 ± 0.59	4.02 ± 0.36	3.26 ± 0.55	13.54 ± 0.39	10.76 ± 0.09	10.68 ± 1.01
theaspirane b ^#^	1321	3.80 ± 0.71	4.55 ± 0.34	3.52 ± 0.73	11.69 ± 0.24	12.65 ± 0.2	8.62 ± 1.49
cyclosativene	1373	-	-	tr	0.21 ± 0.01	0.24 ± 0.02	0.35 ± 0.08
α-copaene	1382	0.22 ± 0.06	tr	0.63 ± 0.08	0.55 ± 0.06	0.84 ± 0.04	0.68 ± 0.11
(*E*)-β-damascenone	1384	36.77 ± 0.12 _b_	33.66 ± 2.62 _c_	21.16 ± 1.52 _b,c_	22.97 ± 1.17	23.78 ± 0.55	13.90 ± 2.42
tetradecane	1400	5.79 ± 0.01 _b_	4.65 ± 0.06	2.86 ± 0.68 _b_	4.17 ± 0.14	4.18 ± 0.65	7.93 ± 2.71
(*E*)-β-damascone	1417	1.29 ± 0.16	1.78 ± 0.02	1.44 ± 0.23	0.91 ± 0.05	1.32 ± 0.29	1.22 ± 0.13
β-caryophyllene	1427	4.06 ± 0.06	4.48 ± 0.58	3.88 ± 0.19	6.44 ± 0.14	7.95 ± 1.34	2.99 ± 0.98
dihydrodehydro-β-ionone	1431	7.75 ± 0.48 _a,b_	3.53 ± 0.32 _a_	3.73 ± 0.42 _b_	1.91 ± 0.2	2.34 ± 0.36	2.34 ± 0.43
(*E*)-geranyl acetone	1461	0.87 ± 0.02	0.98 ± 0.02	0.89 ± 0.1	tr	0.67 ± 0.57	0.38 ± 0.2
α-humulene	1464	1.33 ± 0.04 _b_	1.40 ± 0.02 _c_	2.00 ± 0.17 _b,c_	0.7 ± 0.05	1.04 ± 0.14	0.87 ± 0.25
β-ionone	1488	4.4 ± 0.29	5.14 ± 0.46	3.32 ± 0.4	4.66 ± 0.49	4.75 ± 0.1	4.92 ± 0.18
α-muurolene	1512	0.18 ± 0.06	-	0.20 ± 0.03	tr	tr	tr
α-farnesene	1514	tr	0.35 ± 0.06 _c_	0.98 ± 0.22 _c_	0.22 ± 0.05 _b_	0.36 ± 0.001	0.56 ± 0.1 _b_
δ-cadinene	1527	0.28 ± 0.07	0.56 ± 0.02	tr	0.31 ± 0.02	0.29 ± 0.04	0.27 ± 0.01
kessane	1542	-	-	-	0.16 ± 0.02	0.25 ± 0.02	0.20 ± 0.05
liguloxide	1547	0.86 ± 0.02 _b_	0.82 ± 0.01 _c_	0.59 ± 0.02 _b,c_	1.80 ± 0.09 _a,b_	2.60 ± 0.03 _a_	2.95 ± 0.12 _b_
(*E*)-nerolidol	1575	0.55 ± 0.07	0.94 ± 0.28	1.79 ± 0.19	0.36 ± 0.03 _a,b_	1.47 ± 0.22 _a_	2.24 ± 0.57 _b_
caryophyllene oxide	1594	1.52 ± 0.01	1.29 ± 0.09	1.24 ± 0.09	0.36 ± 0.02 _b_	0.56 ± 0.12	0.74 ± 0.13 _b_
(*E*)-2-hexenyl benzoate	1599	0.92 ± 0.02 _a_	0.22 ± 0.02 _a_	-	0.57 ± 0.15	0.39 ± 0.06	0.65 ± 0.08
hexadecane	1606	1.55 ± 0.25	0.75 ± 0.13	1.12 ± 0.42	1.42 ± 0.14	1.51 ± 0.004	3.60 ± 1.31
	Alcohols	2.41	3.05	2.58	4.39	3.46	4.27
	Aldehydes	11.55	15.83	34.79	13.58	9.34	20.70
	Ketones	52.05	46.30	31.25	31.69	33.58	23.38
	Esters	2.46	1.87	1.89	2.25	1.60	2.29
	Aromatic hydrocarbones	1.39	0.66	0.82	0.53	0.18	0.27
	Saturated hydrocarbons	7.34	5.40	3.98	5.59	5.69	11.53
	Monoterpenes	2.46	3.28	2.49	1.54	1.62	2.23
	Sesquiterpenes	9.08	9.90	11.43	11.21	15.70	11.96
	Heterocyclic compounds	8.87	9.72	7.71	27.30	24.45	20.43
	Pyridines	-	1.78	0.59	-	3.43	-
Total identified (%)		96.90	96.86	96.73	97.37	98.21	96.47

Results are expressed as mean ± SE. Kovats indices (KI) are determined on the VF-5MS capillary column; -, not detected; tr, traces (<0.01%). Compounds are identified by mass spectra and KI comparison with NIST and Wiley libraries, as well as literature values [34,35,36,37]. Statistical analyses are performed by one-way ANOVA analysis, with or without Welch corrections. Means were compared by Tukey’s test if equal variance could be assumed or by Dunett T3’s test if equal variance could not be assumed. Mean values with the same letter differ significantly (*p* ≤ 0.05). theaspirane ^#^—correct isomer could not be determined.

## Data Availability

The original contributions generated for this study are included in the article/Supplementary Material; the data presented in this study are available on request from the corresponding author.

## Supplementary Materials

The following are available online, Figure S1: GC-MS total ion chromatogram of olive leaves essential oil from cv. Frantoio harvested on 12 September, Figure S2: GC-MS total ion chromatogram of olive leaves essential oil from cv. Lastovka harvested on 12th September, Figure S3: GC-MS total ion chromatogram of olive leaves essential oil from cv. Leccino harvested on 12 September, Figure S4: GC-MS total ion chromatogram of olive leaves essential oil from cv. Oblica harvested on 12 September, Figure S5: Principal component analysis of the surface area, color variability, total phenols, and the volatiles in the amount larger than 2% from cvs. Lastovka (LAST), Oblica (OBL), Leccino (LECC) and Frantoio (FRANT) at different harvest periods (1–3), Figure S6: Principal component analysis of the surface area and color variability from cvs. Lastovka (LAST), Oblica (OBL), Leccino (LECC) and Frantoio (FRANT) at different harvest periods (1–3), Figure S7: Principal component analysis of the total phenols and the volatiles in the amount larger than 2% from cvs. Lastovka (LAST), Oblica (OBL), Leccino (LECC), and Frantoio (FRANT) at different harvest periods (1–3), Table S1: *p*-values obtained by Post Hoc One-Way ANOVA analysis of leaf area, color intensity, and total phenolic content from cvs. Lastovka, Oblica, Leccino and Frantoio at different harvest periods, Table S2: *p*-values obtained by one-way ANOVA of volatile compositions from cvs. Lastovka, Oblica, Leccino and Frantoio at different harvest periods, Table S3: Principle component analysis. Varimax with Kaiser Normalization was used for the rotation method in the rotated component matrix.

## Sample Availability

Samples of the leaves and essential oils are available from the authors.
