# Supplementary material for: Essential Oil Volatile Fingerprint Differentiates Croatian cv. Oblica from Other Olea europaea L. Cultivars
Source: Molecules. 2021 Jun 9;26(12):3533. doi: 10.3390/molecules26123533 (PMC8226588; doi:10.3390/molecules26123533)
Supplement: Supplementary file 1 [file molecules-26-03533-s001.zip › Supplementary Figures_corr.pdf]

Supplementary Figures 1-7

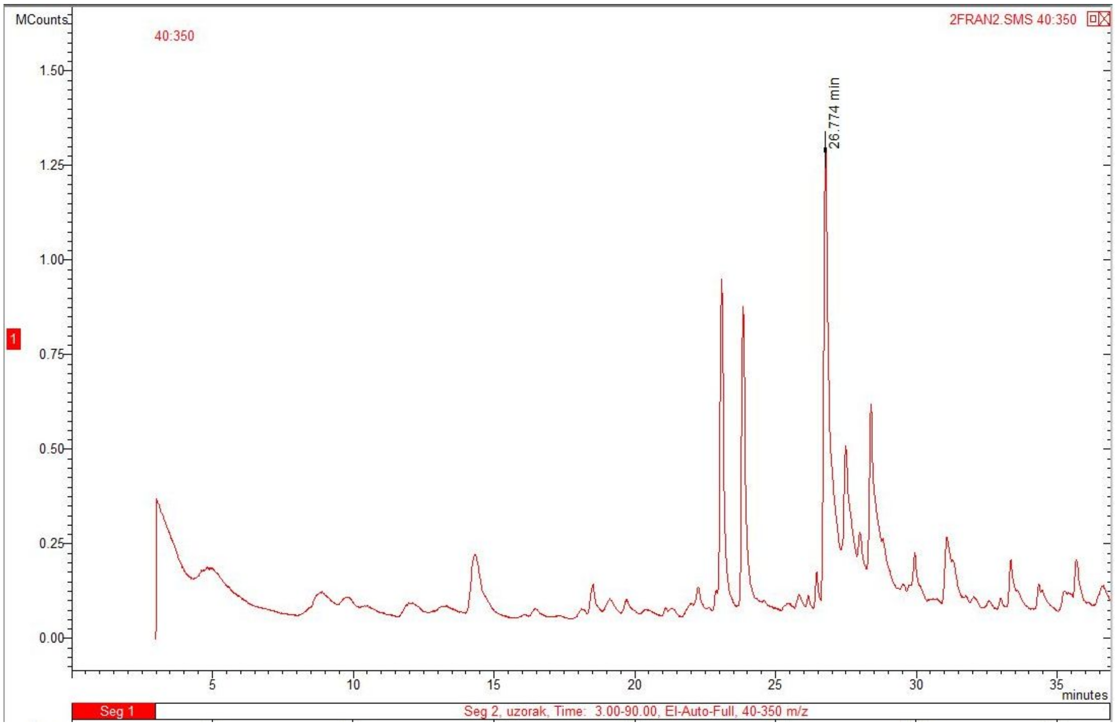

Suppl. Fig. 1. GC-MS total ion chromatogram of olive leaves essential oil from cv. Frantoio harvested on 12th September

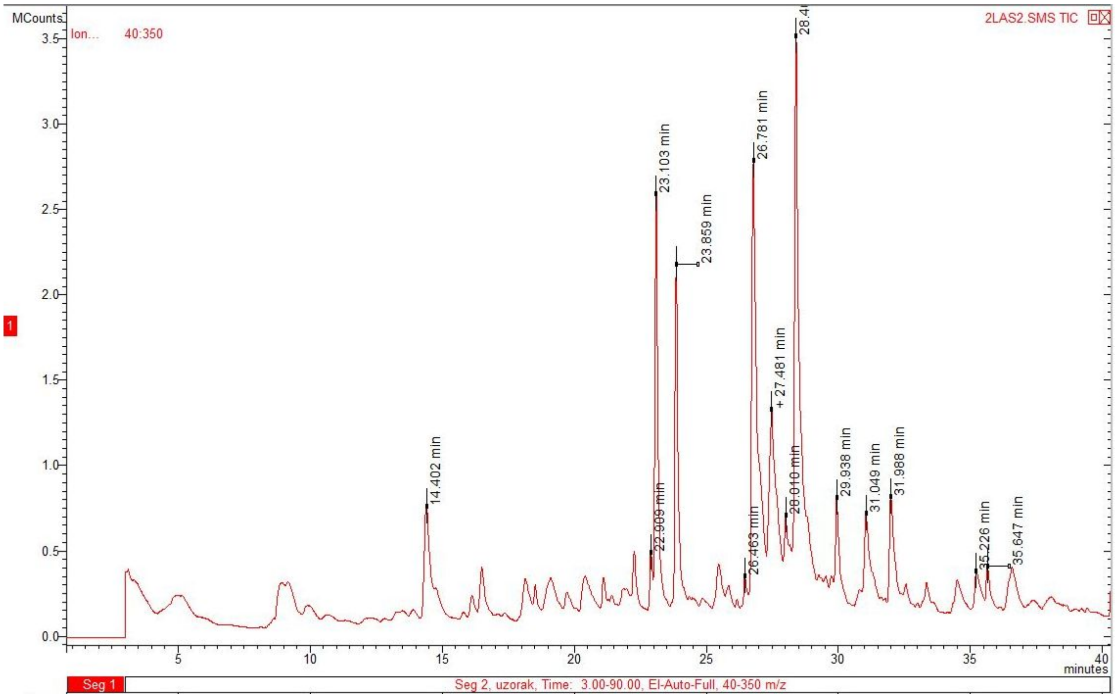

Suppl. Fig. 2. GC-MS total ion chromatogram of olive leaves essential oil from cv. Lastovka harvested on 12th September

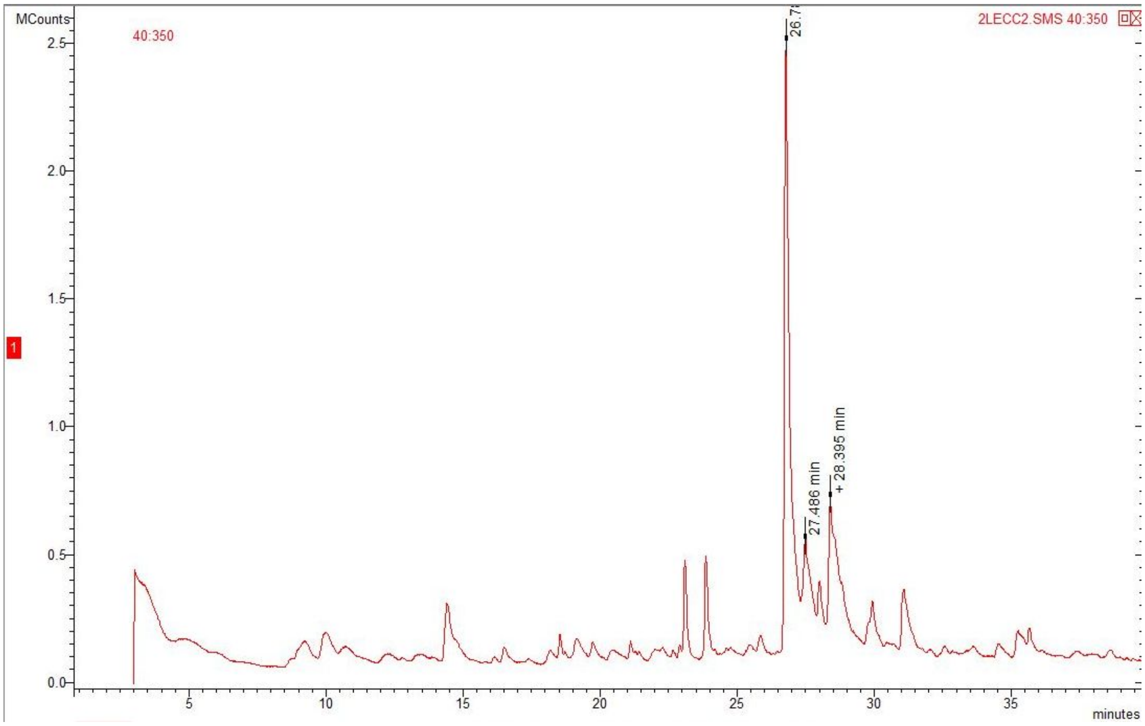

Suppl. Fig. 3. GC-MS total ion chromatogram of olive leaves essential oil from cv. Leccino harvested on 12th September

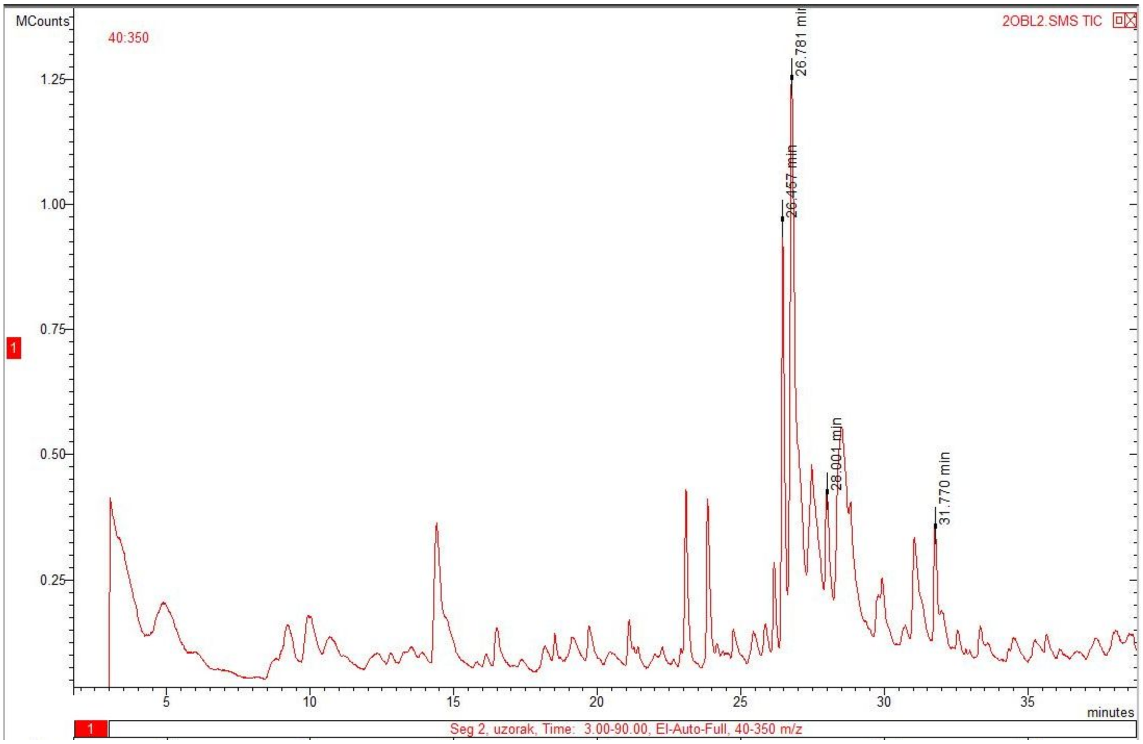

Suppl. Fig. 4. GC-MS total ion chromatogram of olive leaves essential oil from cv. Oblica harvested on 12th September

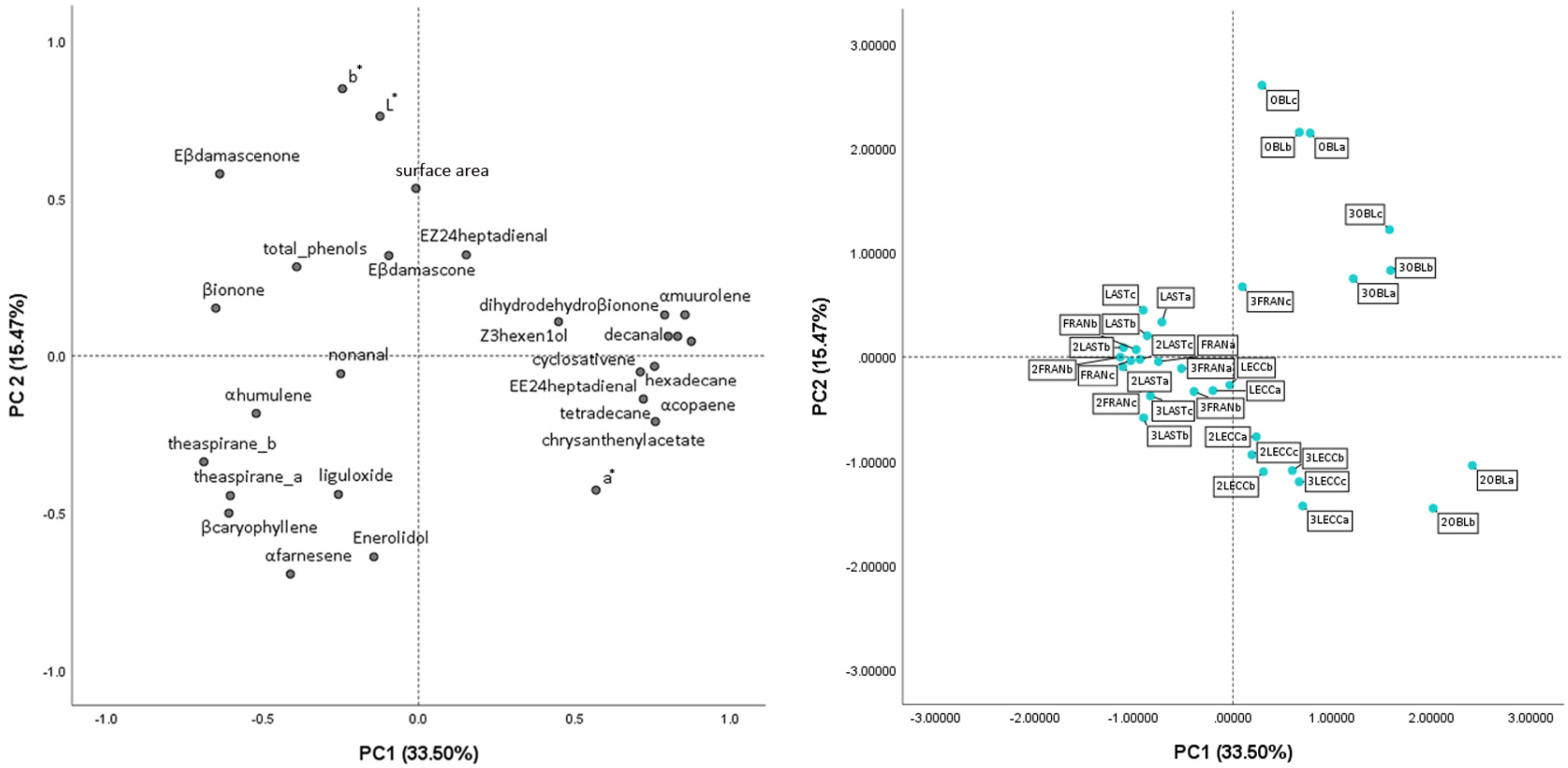

Suppl. Fig. 5. Principal component analysis of the surface area, color variability, total phenols, and the volatiles in the amount larger than 2% from cvs. Lastovka (LAST), Oblica (OBL), Leccino (LECC), and Frantoio (FRANT) at different harvest periods (1-3)

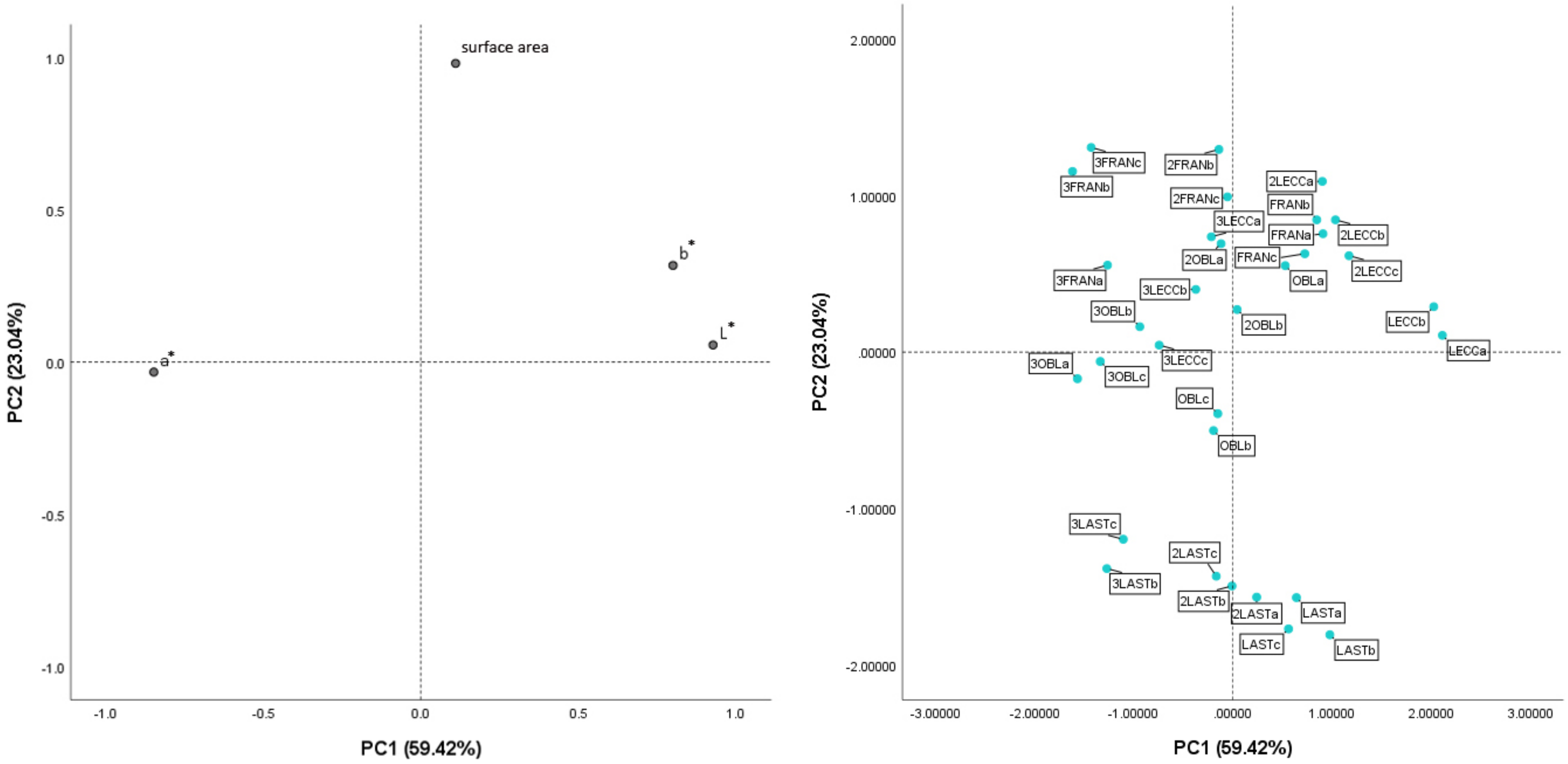

Suppl. Fig. 6. Principal component analysis of the surface area and color variability from cvs. Lastovka (LAST), Oblica (OBL), Leccino (LECC), and Frantoio (FRANT) at different harvest periods (1-3).

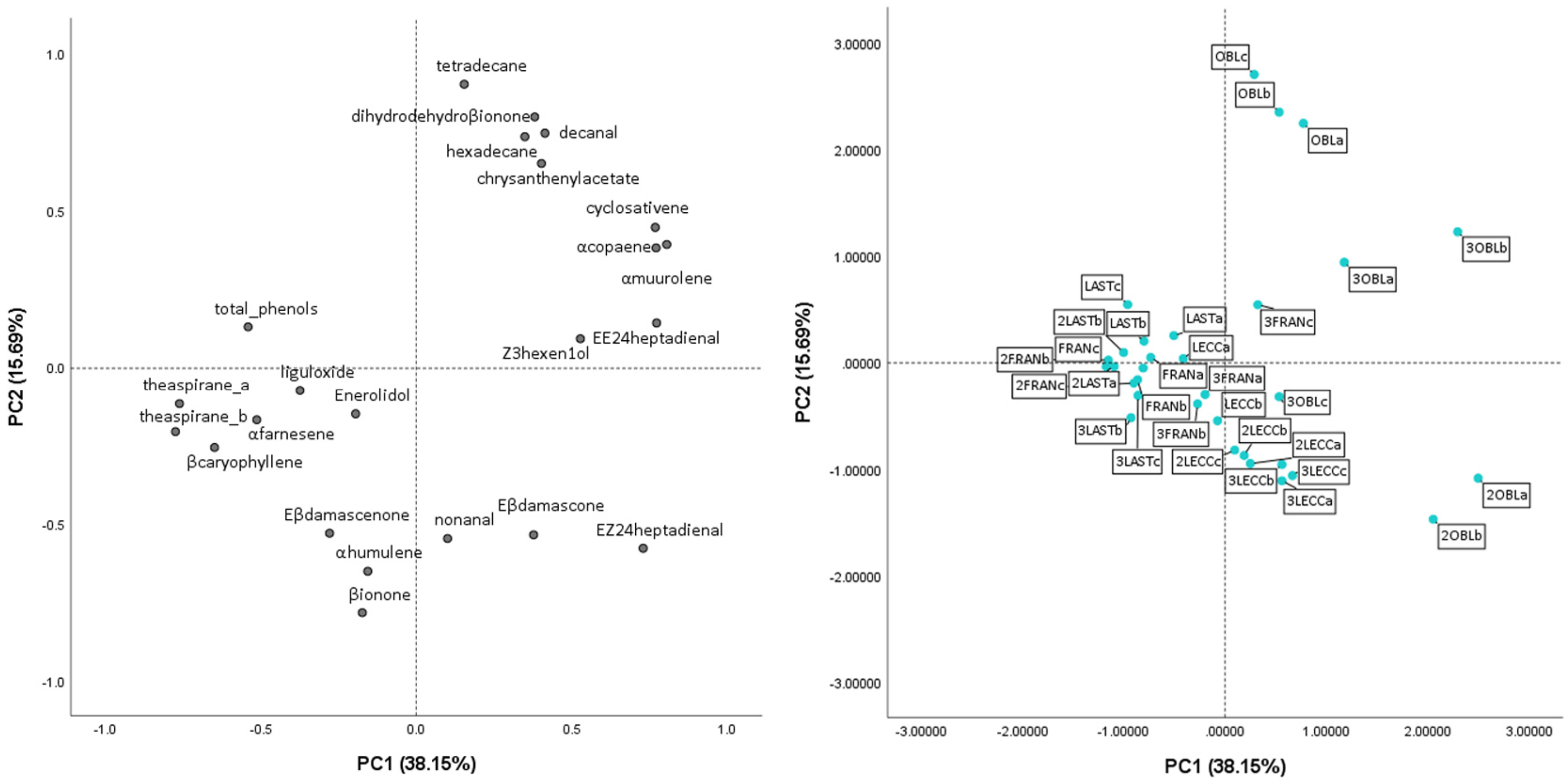

Suppl. Fig. 7. Principal component analysis of the total phenols and the volatiles in the amount larger than 2% from cvs. Lastovka (LAST), Oblica (OBL), Leccino (LECC), and Frantoio (FRANT) at different harvest periods (1-3)
