# Supplementary material for: Essential Oil Volatile Fingerprint Differentiates Croatian cv. Oblica from Other Olea europaea L. Cultivars
Source: Molecules. 2021 Jun 9;26(12):3533. doi: 10.3390/molecules26123533 (PMC8226588; doi:10.3390/molecules26123533)
Supplement: Supplementary file 1 [file molecules-26-03533-s001.zip › Supplementary Tables_corr.pdf]

Supplementary Table 1. *p*-values obtained by Post Hoc One-Way ANOVA analysis of leaf area, color intensity, and total phenolic content from cvs. Lastovka, Oblica, Leccino, and Frantoio at different harvest periods

	leaf area (cm^2)			***color intensity (D65)***									***total phenolic content (mg kg^{-1})***		
				L*			***a****			***b****					
**Harvest periods**			***p*-values**			***p*-values**			***p*-values**			***p*-values**			***p*-values**
August	*Lastovka*	*Oblica*	$2.22x10^{-14}$**	*Lastovka*	*Oblica*	$3.54x10^{-5}$*	*Lastovka*	*Oblica*	NS**	*Lastovka*	*Oblica*	0.009**	*Lastovka*	*Oblica*	NS*
		Leccino	$5.65x10^{-13}$**		*Leccino*	$1.73x10^{-11}$*		*Leccino*	$1.69x10^{-11}$**		*Leccino*	$8.67x10^{-12}$**		*Leccino*	NS*
		Frantoio	$2.83x10^{-14}$**		*Frantoio*	$4.42x10^{-8}$*		*Frantoio*	$1.78x10^{-11}$**		*Frantoio*	0.001**		*Frantoio*	NS*
	Oblica	*Leccino*	0.002**	*Oblica*	*Leccino*	$1.75x10^{-11}$*	*Oblica*	*Leccino*	$1.07x10^{-11}$**	*Oblica*	*Leccino*	$1.78x10^{-11}$**	*Oblica*	*Leccino*	NS*
		Frantoio	$5.69x10^{-14}$**		*Frantoio*	NS*		*Frantoio*	$1.47x10^{-11}$**		*Frantoio*	$2.59x10^{-9}$**		*Frantoio*	NS*
	Leccino	*Frantoio*	$1.54x10^{-13}$**	*Leccino*	*Frantoio*	$1.69x10^{-11}$*	*Leccino*	*Frantoio*	$1.66x10^{-11}$**	*Leccino*	*Frantoio*	$1.33x10^{-11}$**	*Leccino*	*Frantoio*	NS*
September	*Lastovka*	*Oblica*	$4.96x10^{-8}$**	*Lastovka*	*Oblica*	$1.62x10^{-11}$**	*Lastovka*	*Oblica*	$1.54x10^{-11}$**	*Lastovka*	*Oblica*	$6.03x10^{-12}$**	*Lastovka*	*Oblica*	NS**
		Leccino	$4.22x10^{-15}$**		*Leccino*	$1.02x10^{-11}$**		*Leccino*	$4.81x10^{-12}$**		*Leccino*	$1.78x10^{-12}$**		*Leccino*	NS**
		Frantoio	$1.78x10^{-7}$**		*Frantoio*	NS**		*Frantoio*	$1.34x10^{-11}$**		*Frantoio*	$8.62x10^{-12}$**		*Frantoio*	NS**
	Oblica	*Leccino*	$2.447x10^{-11}$**	*Oblica*	*Leccino*	$1.46x10^{-11}$**	*Oblica*	*Leccino*	$1.73x10^{-8}$**	*Oblica*	*Leccino*	$2.1x10^{-6}$**	*Oblica*	*Leccino*	NS**
		Frantoio	$3.90x10^{-13}$**		*Frantoio*	$1.60x10^{-11}$**		*Frantoio*	0.0001**		*Frantoio*	$6.16x10^{-8}$**		*Frantoio*	NS**
	Leccino	*Frantoio*	$2.18x10^{-8}$**	*Leccino*	*Frantoio*	$1.03x10^{-11}$**	*Leccino*	*Frantoio*	NS**	*Leccino*	*Frantoio*	$1.35x10^{-11}$**	*Leccino*	*Frantoio*	NS**
October	*Lastovka*	*Oblica*	$4.06x10^{-6}$**	*Lastovka*	*Oblica*	0.142**	*Lastovka*	*Oblica*	$6.41x10^{-5}$**	*Lastovka*	*Oblica*	$9.04x10^{-10}$**	*Lastovka*	*Oblica*	0.004*
		Leccino	$3.90x10^{-13}$**		*Leccino*	$2x10^{-13}$**		*Leccino*	$1.75x10^{-13}$**		*Leccino*	$1.79x10^{-13}$**		*Leccino*	0.001*
		Frantoio	$5.26x10^{-14}$**		*Frantoio*	NS**		*Frantoio*	$1.78x10^{-9}$**		*Frantoio*	NS**		*Frantoio*	0.01*
	Oblica	*Leccino*	$4.12x10^{-13}$**	*Oblica*	*Leccino*	$3.2x10^{-12}$**	*Oblica*	*Leccino*	$1.65x10^{-11}$**	*Oblica*	*Leccino*	NS**	*Oblica*	*Leccino*	NS*
		Frantoio	$5.44x10^{-15}$**		*Frantoio*	0.006**		*Frantoio*	NS**		*Frantoio*	$1.96x10^{-11}$**		*Frantoio*	NS*
	Leccino	*Frantoio*	$7.74x10^{-6}$**	*Leccino*	*Frantoio*	$6.36x10^{-13}$**	*Leccino*	*Frantoio*	$1.16x10^{-11}$**	*Leccino*	*Frantoio*	$5.21x10^{-12}$**	*Leccino*	*Frantoio*	NS*

Cultivars			*p*-values			*p*-values			*p*-values			*p*-values			*p*-values
Lastovka	August	September	$7.69x10^{-5*}$	August	September	$1.65x10^{-11**}$	August	September	$1.02x10^{-9**}$	August	September	$1.19x10^{-6**}$	August	September	0.018^{*}
		October	NS^{*}		October	$1.83x10^{-12**}$		October	$5.10x10^{-9**}$		October	$5.1x10^{-9**}$		October	NS^{*}
	September	October	$3.58x10^{-6*}$	September	October	$4.93x10^{-12**}$	September	October	$1.38x10^{-12**}$	September	October	0.0011^{**}	September	October	NS^{*}
Oblica	August	September	0.01^{*}	August	September	$1.73x10^{-11**}$	August	September	$1.04x10^{-11**}$	August	September	$1.61x10^{-11**}$	August	September	NS^{*}
		October	0.001^{*}		October	$4.79x10^{-12**}$		October	$6.10x10^{-12**}$		October	NS^{**}		October	0.043^{*}
	September	October	$6.36x10^{-9*}$	September	October	$7.11x10^{-12**}$	September	October	$1.68x10^{-11**}$	September	October	$8.63x10^{-12**}$	September	October	NS^{*}
Leccino	August	September	$7.98x10^{-9*}$	August	September	$1.52x10^{-11*}$	August	September	0.004^{**}	August	September	$1.74x10^{-11**}$	August	September	0.032^{*}
		October	NS^{*}		October	$1.68x10^{-11*}$		October	$1.76x10^{-11**}$		October	$8.87x10^{-12**}$		October	0.0002^{*}
	September	October	$8.54x10^{-8*}$	September	October	$1.7x10^{-11*}$	September	October	$1.80x10^{-11**}$	September	October	$8.30x10^{-12**}$	September	October	0.003^{*}
Frantoio	August	September	$3.89x10^{-5**}$	August	September	$1.65x10^{-11**}$	August	September	NS^{**}	August	September	NS^{**}	August	September	NS^{**}
		October	NS^{**}		October	$3.77x10^{-12*}$		October	$5.81x10^{-12**}$		October	$2.32x10^{-12**}$		October	0.039^{**}
	September	October	$1.83x10^{-6**}$	September	October	$6.66x10^{-12*}$	September	October	$7.99x10^{-12**}$	September	October	$1.45x10^{-12**}$	September	October	NS^{**}

p-values are obtained by one-way ANOVA analysis, with or without Welch corrections. Means were compared by Tukey's test (*) if equal variance could be assumed, or by Dunett T3's test (**). If equal variance could not be assumed. Statistical significant test result is considered for $p \leq 0.05$. NS-non significant.

Supplementary Table 2. *p*-values obtained by one way ANOVA of volatile compositions from cvs. Lastovka, Oblica, Leccino, and Frantoio at different harvest periods

Compound	Lastovka	Oblica	Leccino	Frantoio
	***p*-values**			
(*Z*)-3-hexen-1-ol	0.044**	NS*	NS*	0.047*
heptanal	-	NS*	0.001*	NS*
benzaldehyde	< 0.001**	NS*	0.047*	0.005*
pyridne-3-ethnyl	< 0.001*	-	NS*	-
(*E,Z*)-2,4-heptadienal	NS*	0.004*	NS**	NS*
(*E,E*)-2,4-heptadienal	NS**	NS*	0.001*	0.001*
phenylacetaldehyde	NS**	NS*	NS*	NS*
cis-linalool oxide (furanoid)	0.033**	NS**	-	-
1-octanol	0.038*	-	NS*	0.001*
trans-linalool oxide (furanoid)	NS*	NS**	-	-
p-cymenene	NS**	0.039*	0.04*	-
nonanal	NS*	0.018*	0.002*	0.014*
1,4-dimethyl-δ-3-tetrahydroacetophenone	NS*	0.008*	0.039*	NS*
limonen-4-ol	NS*	NS*	NS*	NS*
decanal	0.03*	0.024*	NS*	NS**
α-terpineol	NS*	NS*	NS**	NS*
methyl salicylate	0.002*	NS*	NS*	NS**
β-cyclocitral	0.031*	NS*	NS**	NS*
chrysanthenyl acetate	NS*	NS*	NS*	NS*
α-ionene	0.021*	NS*	0.012*	NS**
(*E*)-2-decenal	0.006*	-	0.007*	NS*
vitispirane	0.016*	0.039*	0.028*	0.003*
dihydroedulan II	-	NS*	NS*	-
dihydroedulan I	0.004**	-	0.006*	NS**
theaspirane a	0.01*	NS*	NS*	NS*
theaspirane b	0.003*	0.036*	NS*	NS**
cyclosativene	0.019*	NS*	-	NS*
α-copaene	0.021*	NS*	NS**	NS*
(*E*)-β-damascenone	0.026*	NS*	0.006*	NS**
tetradecane	0.001*	0.001*	0.002**	NS*
(*E*)-β-damascone	0.014*	NS*	NS*	NS*
β-caryophyllene	0.001*	NS*	NS*	0.042*
dihydrodehydro-β-ionone	0.035*	0.004*	0.008*	NS*
(*E*)-geranyl acetone	0.001*	-	NS*	NS**
α-humulene	0.007*	NS*	0.010*	NS*
β-ionone	0.023*	0.004*	NS*	NS*
α-muurolene	-	NS*	NS*	NS*
α-farnesene	0.023*	-	0.001*	0.048*
δ-cadinene	0.033*	NS*	NS**	NS*
kessane	-	-	-	NS*
liguloxide	< 0.001*	0.017*	0.008**	0.001*
(*E*)-nerolidol	< 0.001*	NS*	NS*	0.002*
caryophyllene oxide	NS*	NS*	NS*	0.037*
(*E*)-2-hexenyl benzoate	-	-	0.001*	NS*
hexadecane	NS*	0.05*	NS*	NS*

p-values are obtained by one-way ANOVA analysis, with or without Welch corrections. Statistical significant test result is considered for $p \leq 0.05$. - not enough data to perform the analysis. NS-non significant.

Supplementary Table 3. Principle component analysis. Varimax with Kaiser Normalization was used for the rotation method in the rotated component matrix.

Volatile compound	Communalities	Rotated Component Matrix		Component Score Coefficient Matrix	
		Component 1	Component 2	Component 1	Component 2
**volatiles > 2%**					
(*Z*)-3-hexen-1-ol	0.224	0.743	-0.255	0.275	-0.194
(*E,Z*)-2,4-heptadienal	0.844	0.373	0.219	0.119	0.020
(*E,E*)-2,4-heptadienal	0.490	0.489	0.256	0.036	0.005
nonanal	0.315	-0.182	0.099	-0.019	0.026
decanal	0.850	0.399	0.440	-0.002	0.058
chrysanthenyl acetate	0.515	0.377	0.508	0.026	0.119
theaspirane a	0.666	-0.179	-0.914	0.097	-0.269
theaspirane b	0.703	-0.252	-0.909	0.074	-0.255
cyclosativene	0.777	0.871	0.320	0.248	-0.028
α-copaene	0.719	0.864	0.338	0.263	-0.021
(*E*)-β-damascenone	0.406	-0.746	-0.033	-0.254	0.096
tetradecane	0.849	0.209	0.307	-0.082	0.051
(*E*)-β-damascone	0.522	0.113	0.224	-0.027	0.117
β-caryophyllene	0.509	-0.223	-0.312	0.086	-0.027
dihydrodehydro-β-ionone	0.783	0.276	0.608	-0.072	0.144
humulene	0.490	-0.181	0.176	0.020	0.132
β-ionone	0.571	-0.263	-0.523	-0.039	-0.099
α-muurolene	0.789	0.841	0.398	0.239	0.001
α-farnesene	0.308	-0.167	-0.116	0.045	0.038
liguloxide	0.184	-0.038	-0.776	0.001	-0.247
(*E*)-nerolidol	0.050	-0.039	-0.090	-0.057	0.012
hexadecane	0.688	0.291	0.203	-0.065	-0.012
**surface area, color, total phenols and volatiles > 2%**					
(*Z*)-3-hexen-1-ol	0.212	0.448	0.107	0.049	0.021
(*E,Z*)-2,4-heptadienal	0.125	0.152	0.319	0.014	0.075
(*E,E*)-2,4-heptadienal	0.506	0.710	-0.052	0.080	-0.019
nonanal	0.066	-0.250	-0.058	-0.027	-0.011

decanal	0.642	0.799	0.061	0.088	0.007
chrysanthenyl acetate	0.619	0.758	-0.211	0.087	-0.058
theaspirane a	0.563	-0.604	-0.446	-0.063	-0.100
theaspirane b	0.589	-0.689	-0.338	-0.073	-0.074
cyclosativene	0.764	0.873	0.045	0.097	0.002
α-copaene	0.692	0.829	0.061	0.092	0.006
(*E*)-β-damascenone	0.739	-0.638	0.576	-0.077	0.144
tetradecane	0.537	0.719	-0.139	0.082	-0.040
(*E*)-β-damascone	0.110	-0.096	0.317	-0.014	0.077
β-caryophyllene	0.621	-0.608	-0.501	-0.063	-0.113
dihydrodehydro-β-ionone	0.638	0.788	0.129	0.086	0.023
humulene	0.305	-0.521	-0.185	-0.056	-0.039
β-ionone	0.445	-0.650	0.150	-0.074	0.042
α-muurolene	0.744	0.853	0.129	0.094	0.022
α-farnesene	0.651	-0.411	-0.695	-0.039	-0.162
liguloxide	0.262	-0.257	-0.442	-0.024	-0.103
(*E*)-nerolidol	0.430	-0.144	-0.640	-0.010	-0.151
hexadecane	0.572	0.755	-0.034	0.084	-0.016
total phenols	0.232	-0.391	0.282	-0.046	0.071
leaf area	0.282	-0.009	0.531	-0.006	0.127
L*	0.592	-0.124	0.760	-0.021	0.182
a*	0.506	0.568	-0.428	0.068	-0.108
b*	0.777	-0.244	0.847	-0.036	0.204
**surface area and color**					
surface area	0.978	0.110	0.983	-0.145	0.977
L*	0.861	0.926	0.056	0.442	-0.130
a*	0.719	-0.847	-0.033	-0.408	0.138
b*	0.740	0.799	0.318	0.327	0.162
**total phenols and the volatiles > 2%**					
(*Z*)-3-hexen-1-ol	0.286	0.527	0.091	0.091	-0.024
(*E*,*Z*)-2,4-heptadienal	0.864	0.729	-0.578	0.183	-0.177

(*E,E*)-2,4-heptadienal	0.615	0.771	0.141	0.133	-0.034
nonanal	0.309	0.100	-0.547	0.063	-0.120
decanal	0.725	0.413	0.745	0.017	0.119
chrysanthenyl acetate	0.582	0.402	0.648	0.022	0.100
theaspirane a	0.594	-0.762	-0.117	-0.133	0.038
theaspirane b	0.642	-0.775	-0.206	-0.128	0.021
cyclosativene	0.787	0.767	0.445	0.107	0.029
α-copaene	0.737	0.770	0.380	0.113	0.015
(*E*)-β-damascenone	0.359	-0.279	-0.530	-0.009	-0.086
tetradecane	0.834	0.153	0.900	-0.044	0.172
(*E*)-β-damascone	0.428	0.376	-0.535	0.114	-0.140
β-caryophyllene	0.487	-0.649	-0.257	-0.100	0.000
dihydrodehydro-β-ionone	0.779	0.380	0.796	0.006	0.132
humulene	0.448	-0.157	-0.651	0.024	-0.120
β-ionone	0.643	-0.174	-0.783	0.031	-0.146
α-muurolene	0.800	0.804	0.391	0.118	0.015
α-farnesene	0.292	-0.513	-0.168	-0.082	0.007
liguloxide	0.146	-0.374	-0.074	-0.064	0.015
(*E*)-nerolidol	0.061	-0.196	-0.149	-0.024	-0.015
hexadecane	0.659	0.348	0.734	0.006	0.122
total phenols	0.309	-0.541	0.129	-0.111	0.070
